# Alteration of Sarcoplasmic Reticulum Ca^2+^ Release in Skeletal Muscle from Calpain 3-Deficient Mice

**DOI:** 10.1155/2009/340346

**Published:** 2010-03-14

**Authors:** Govindan Dayanithi, Isabelle Richard, Cédric Viero, Elsa Mazuc, Sylvie Mallie, Jean Valmier, Nathalie Bourg, Muriel Herasse, Isabelle Marty, Gérard Lefranc, Paul Mangeat, Stephen Baghdiguian

**Affiliations:** ^1^Department of Cellular Neurophysiology, Institute of Experimental Medicine, Academy of Sciences of the Czech Republic, European Union Centre of Excellence, Videnska 1083, 142 20 Prague 4, Czech Republic; ^2^Départment Biologie Santé, Université Montpellier 2, place Eugène Bataillon, 34095 Montpellier, Cedex 05, France; ^3^Généthon CNRS-UMR8587 LAMBE, 1, rue de l'Internationale, 91000 Evry, France; ^4^Department of Cardiology, Wales Heart Research Institute, School of Medicine, Cardiff University, Heath Park, Cardiff CF14 4XN, UK; ^5^INSERM U583, Institut des Neurosciences de Montpellier, Hôpital St Eloi, 80, rue Augustin Fliche, 34091 Montpellier, Cedex 05, France; ^6^Institut de Recherche en Cancérologie de Montpellier, CRLC Val d'Aurelle-Paul Lamarque, 34298 Montpellier, Cedex 05, France; ^7^Grenoble Institut des Neurosciences, INSERM U836, Equipe Muscle et Pathologies, UJF Site santé-BP170, 38042 Grenoble Cedex 9, France; ^8^Laboratoire d'Immunogénétique Moléculaire, UPR 1142 CNRS-Institut de Génétique Humaine, Université Montpellier 2, place Eugène Bataillon, 34095 Montpellier, Cedex 05, France; ^9^Centre de Recherche de Biochimie Macromoléculaire, Universités Montpellier 2 & 1, 1919 Route de Mende, 34293 Montpellier, Cedex 05, France; ^10^UMR CNRS 5554, Institut des Sciences de l'Evolution, Université Montpellier 2, place Eugène Bataillon, 34095 Montpellier, Cedex 05, France

## Abstract

Mutations of Ca^2+^-activated proteases (calpains) cause muscular dystrophies. Nevertheless, the specific role of calpains in Ca^2+^ signalling during the onset of dystrophies remains unclear. We investigated Ca^2+^ handling in skeletal cells from calpain 3-deficient mice. [Ca^2+^]_*i*_ responses to caffeine, a ryanodine receptor (RyR) agonist, were decreased in −/− myotubes and absent in −/− myoblasts. The −/− myotubes displayed smaller amplitudes of the Ca^2+^ transients induced by cyclopiazonic acid in comparison to wild type cells. Inhibition of L-type Ca^2+^ channels (LCC) suppressed the caffeine-induced [Ca^2+^]_*i*_ responses in −/− myotubes. Hence, the absence of calpain 3 modifies the sarcoplasmic reticulum (SR) Ca^2+^ release, by a decrease of the SR content, an impairment of RyR signalling, and an increase of LCC activity. We propose that calpain 3-dependent proteolysis plays a role in activating support proteins of intracellular Ca^2+^ signalling at a stage of cellular differentiation which is crucial for skeletal muscle regeneration.

## 1. Introduction

Calpains are intracellular nonlysosomal cysteine proteases whose functions are regulated by Ca^2+^ (see [1] for review). These proteins indeed display one Ca^2+^-binding domain on each of the large and small subunits [2]. The physiological roles of the calpains are not yet fully understood but as proteases, they may regulate important cellular functions. In particular, ubiquitous calpains have been implicated in a wide variety of processes including apoptosis, myogenic differentiation, cellular division and fusion [1]. Calpains have been shown to play regulatory roles in other cells, in which they can influence gene expression through the cleavage of specific transcription factors, affecting cell viability by controlling apoptosis, and modulating other cell processes through the cleavage of specific kinases and ion channels (reviewed by Carafoli and Molinari; [3]). It was demonstrated that the absence of the skeletal muscle specific calpain 3 (from the corresponding gene *capn3*) causes limb girdle muscular dystrophy type 2A (LGMD2A) [4], a disease that has been linked to a significant level of apoptotic fibres [5]. To better understand the function of calpain 3 and the pathophysiological mechanisms of LGMD2A, an adequate model was generated by gene targeting [6]. The pathological process due to calpain 3 deficiency is associated with alterations in membrane permeability [6] suggesting the possible existence of a perturbation in homeostasis, especially in the intracellular Ca^2+^ concentration ([Ca^2+^]*_i_*) during muscular dystrophy.

The identification from skeletal muscle of a 94 kDa protein that possesses thiol-dependent proteolytic activity specifically directed against the skeletal muscle ryanodine receptor (RyR) has several implications for the pathogenesis of LGMD2A [7]. In particular, it makes it possible that the dysregulation of skeletal muscle functions is, at least in part, a consequence of the lack of RyR regulation by calpain 3. 

Indeed, RyR, also referred to as Ca^2+^-release channel, is the key protein responsible for the extremely rapid movement of Ca^2+^ (millions of ions per second) from the internal stores named the sarcoplasmic reticulum (SR) to the cytosol during the process of excitation-contraction coupling. The latter is described by a plasma membrane depolarisation coming from motor neurones and activating dihydropyridine receptors or L-type Ca^2+^ channels (LCCs) mechanically coupled to the skeletal RyR isoform 1. In turn, Ca^2+^ release from RyR causes the contractile filaments to slide along one another, thus triggering the twitch of the muscle cell. Then Ca^2+^ has to be removed from the cytosol by extrusion through the Na^+^/Ca^2+^ exchanger and by reuptake into the SR by the sarcoplasmic/endoplasmic reticulum Ca^2+^ ATPase (SERCA) pump. Hence Ca^2+^ cycling is a fine-tuned process that requires strong control of its homeostasis. 

To test the hypothesis of RyR modulation by calpain 3, we investigated the effect of caffeine, a potent activator of RyR, and cyclopiazonic acid (CPA), an inhibitor of the SERCA pump, on [Ca^2+^]_*i*,_ which controls the excitation-contraction coupling in muscle. We measured [Ca^2+^]*_i_* in primary cultures of normal and *capn3*-deficient mice skeletal muscle cells by single cell fast fluorescence microspectrofluorometry. In vivo, satellite cells are activated into myoblasts, proliferate and fuse to form myotubes to repair damaged muscle fibres. By comparing myoblasts and myotubes in culture, we aimed to investigate the different steps involved in the process of regeneration. Our pharmacological approach enabled us to decipher the mechanisms leading to an impairment of Ca^2+^ release in skeletal muscle when calpain 3 is absent, as it might be the case in LGMD2A patients. 

## 2. Material and Methods

### 2.1. Generation of *capn3*-Deficient Mice

The production of *capn3* −/− mice was carried out according to the well established procedure published in 2000 by Richard and collaborators [6].

### 2.2. Primary Skeletal Muscle Cell Culture

Muscles were excised from the upper and lower legs of adult mice, and proliferating satellite cells were isolated from these muscles by pronase digestion as previously described [8]. Cells were seeded in gelatine-coated (0.5%) glass bottom culture dishes (HBSt or GWSt-3522 series; 22 mm diameter, 0.17 mm thickness; WillCo Wells BV- **A**msterdam, Netherlands) at 2 × 10^3^ cells per cm^2^ in Dulbecco's Modified Eagle Medium (DMEM) containing 20% foetal calf serum, and incubated at 37°C in 7.5% CO_2_. Cells were continuously grown in this medium, which was replaced at day 3 and then every 4 days. Proliferating satellite cells were kept in culture for up to 11 days.

### 2.3. Indirect Immunofluorescence

Indirect immunofluorescence was done as described by Martin et al. in 1997 [9] using appropriate dilutions of primary and secondary fluorescent antibodies. The samples were observed with a Leica TCS 4D confocal microscope.

### 2.4. Dye Loading and Measurement of [Ca^2+^]_*i*_

Dye loading and intracellular free calcium measurements ([Ca^2+^]*_i_*) were performed as described [10–12]. The culture dishes were rinsed and incubated with 2.5 *μ*M fura-2-AM and 0.05% w/v Pluronic F-127 (Molecular Probes, Inc USA) in Locke Locke's buffer (in mM): NaCl 140; KCl 5; MgCl_2_ 1.2; CaCl_2_ 2.2; glucose 10; HEPES-Tris 10; pH 7.25) at 34°C for 45 min. Subsequently, loaded cells were rinsed with Locke's buffer, and fluorescence measurements were carried out in buffers kept at 35–37°C throughout the time course of the experiment. Calcium-free medium (EGTA-buffer) contained (mM): EGTA, 2; NaCl, 140; KCl, 5; glucose, 10; KCl, 5; MgCl_2_, 1; and HEPES 10; pH 7.4. In this EGTA buffer, free Ca^2+^ was adjusted to 100 nM (which corresponds to the resting [Ca^2+^]*_i_* as determined by preliminary fura-2 measurements). [Ca^2+^]*_i_* levels in single cells were measured using the FFP photometer system (Zeiss, Oberkochen, Germany) based on an inverted microscope (Axiovert-100) equipped with epifluorescence. Band pass filters (340/10 nm and 380/10 nm) were alternately positioned with a filter wheel, and the cells were excited through an oil-immersion objective (Zeiss-plan Neofluar ×100, 1.3 n.a). With fluorescence values corrected for background and dark current, [Ca^2+^]*_i_* was calculated from the ratio between 340 and 380 nm recordings. For the *in vitro* calibration of [Ca^2+^]*_i_* measurements based on the procedure described by Grynkiewicz et al. [13], we used Ca^2+^-EGTA buffer containing (mM): NaCl 140; MgCl_2_ 2; glucose 10; HEPES 10 at pH = 7.25 adjusted with Tris-HCl. Various standards were used for system calibration as described previously [13].

### 2.5. Drugs

Unless otherwise stated, all chemicals were purchased from Sigma-France. Concentrated stock DMSO solutions of cyclopiazonic acid (CPA) and ryanodine (Alomone Labs, Israel) were stored at −20°C. Caffeine (Almone Labs, Isreal) was dissolved directly in the working buffer at appropriate concentrations. Test solutions were prepared daily using aliquots from frozen stocks to obtain the working concentrations.

### 2.6. Drug Application

The control and test solutions were applied using a multiple capillary perfusion system (200 *μ*m inner diameter capillary tubing, flow rate 500 *μ*l/min) placed close to the cell tested (<0.5 mm). Each capillary was fed by a reservoir 50 cm above the bath. Switching the opening from one capillary to the next made complete solution changes. After each application, the cells were washed with Locke's buffer. Preincubation with inhibitory substances was carried out in a 500 *μ*L bath containing the inhibitors diluted in Locke's buffer.

### 2.7. Data Analysis and Statistical Methods

The results are expressed as mean ± S.D or means ± S.E.M. The number of sample size (*n*) given is the number of cells tested with the same protocol (control, test drug, recovery) for each group. The figures (traces) show on-line measurements of the [Ca^2+^]*_i_* levels before and after the application of test substances, while bar diagrams and numerical data are given as mean ± S.E.M. and represent the peak amplitude of the [Ca^2+^]*_i_* increase. Depending on the data, the results were analysed using ANOVA or Mann-Whitney Rank Sum test. Unless otherwise stated, differences were considered statistically significant if  *P* < 0.05.

### 2.8. Western Blot

Muscle samples were homogenized in 200 mM sucrose, 0.4 mM CaCl_2_, 20 mM HEPES (pH 7.4), 200 *μ*M phenylmethylsulfonylfluoride (PMSF), 1 mM diisopropylfluorophosphate (DFP). Protein samples were denatured 30 min at room temperature and subjected to SDS-PAGE on 4%–15% acrylamide gradient gels and electro-transferred to Immobilon membranes for 3 hours at 0.8 A. The membrane was blocked with 4% nonfat dry milk in PBS, 0.1% Tween 20 (PBS-T) for 30 minutes at room temperature and then incubated overnight at 4°C with a polyclonal rabbit antibody against RyR diluted 1/10 000 [14]. After washing in PBS-T, the membrane was incubated for 3 hours at room temperature with antirabbit secondary antibodies (Jackson ImmunoResearch Laboratories) coupled to horseradish peroxidase. Revelation was carried out with a chemiluminescent reagent (Western lightning Chemiluminescence reagent plus, Perkin Elmer). Band intensities were quantified using ImageJ software (National Institutes of Health, Bethesda, MD, USA).

### 2.9. RT-PCR Analysis

Total RNA was extracted from muscles by the Trizol method (Invitrogen) after pulverization using a Fast Prep FP120 apparatus (Bio101). Residual DNA was removed from the samples using the Free DNA kit (Ambion). One *μ*g of RNA was reverse-transcribed using random hexamers according to the protocol “Superscript II first strand synthesis system for RT-PCR” (Invitrogen). PCR was carried out on 1/20 of the reaction with 0.2 *μ*M of each primer. Three primer pairs were designed for* capn3 *in order to cover the regions of alternative splicing [4] as follows:

 p94sys3: forward 5′-TTCACCAAATCCAACCACCG-3′ and reverse 5′-ACTCCAAGAACCGTTCCACT-3′;
 p94sys5: forward 5′-AGACAAAGATGAGAAGGCCC-3′ and reverse 5′-GCCGATCCACAGAGATTGTA-3′;
 p94sys6: forward 5′-GACAGAGCACACAGCAACAA-3′ and reverse 5′-GTTGGCTGTTGAGATGGAAG-3′.

PCR products were separated by agarose gel electrophoresis and stained with ethidium bromide. Band intensities were quantified using ImageJ software (National Institutes of Health, Bethesda, MD, USA).

### 2.10. Caspase 3 Activity

To determine and quantify caspase 3 function, we used the PhiPhiLux-G_2_D_2_ substrate kit (OncoImmunin, Inc., MD, USA). Briefly, the substrate is coupled to a fluorophore and when cleaved specifically by caspase 3, the fluorescence can be detected, measured and analysed (excitation and emission peaks are 552 and 580 nm, resp.).

## 3. Results

### 3.1. Morphological and Molecular Characterisation of Living Myoblasts and Myotubes

**Figure 1:**
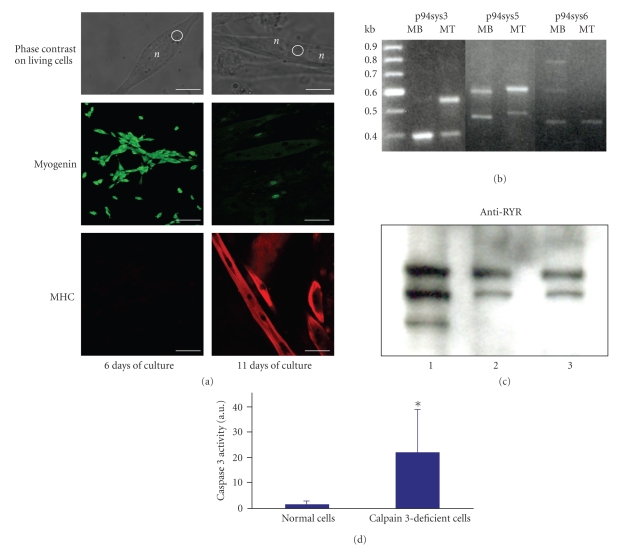
Morphological and molecular features of skeletal muscle cells used throughout this study. (a) Phase contrast and fluorescence micrographs of murine skeletal muscle cells in primary culture. Typical morphology of the living cells (left upper panel: myoblasts; right upper panel: myotubes) used for calcium measurements observed by phase contrast microscopy. Circles indicate the region of drug application and monitoring of [Ca^2+^]*_i_*. Immunological staining of myogenin on myoblasts (left middle panel) and myotubes (right middle panel) was visualized by confocal microscopy using a FITC-labelled secondary antibody (green fluorescence). Myosine Heavy Chain (MHC) was similarly observed in myoblasts (left lower panel) and myotubes (right lower panel) using a TRITC-labelled secondary antibody (red fluorescence). Myoblasts and myotubes were obtained after 6 or 11 days in culture, respectively. (b) Detection of calpain 3-mRNA in wild type (+/+) myoblasts and myotubes by RT-PCR. Gel electrophoresis of the RT-PCR reactions obtained using the primer pairs p94sys3, p94sys5 and p94sys6 (see Section 2) on murine myoblast (MB) or myotube (MT) mRNA. (c) Detection of the ryanodine receptor in skeletal muscle from normal and *capn3*-deficient mice. Muscle from normal (Lane 3) and *capn3*-deficient mice (Lane 2) were extracted and left 30 min at room temperature to allow the cleavage of RyR and were then subjected to SDS-PAGE. Human muscle was used as control (Lane1). No difference in the cleavage pattern was observed, indicating that the partial cleavage of RyR also occurs in the absence of calpain 3 in this biochemical assay. (d) Measurement of caspase 3 activity in wild type and *capn3*-deficient myoblasts. The graph displays the levels of substrate cleavage expressed as means ± S.D. in arbitrary units. The results are based on 4 different experiments. The differences in the median values among the two groups are greater than would be expected by chance; there is a statistically significant difference (*P* = 0.029), as indicated by a Mann-Whitney Rank Sum test.

We first characterised the differentiated states of the skeletal muscle primary cultures used in this study by the expression of specific differentiation markers. At day 6 of culture, mononuclear cells from both wild type (Figure 1(a), left upper panel) and *capn3*-deficient (not shown) mice were uniformly positive for myogenin (Figure 1(a), left medium panel) and negative for MHC (Figure 1(a), left lower panel) confirming that they were differentiated myoblasts. After 11 days in culture, cells became multinucleated (Figure 1(a), right upper panel), and the myogenin staining was strongly reduced (Figure 1(a) right medium panel); in contrast, cells expressed prominent MHC staining (Figure 1(a) right lower panel). Therefore, at that time most myoblasts had fused into myotubes, showing a serum-induced further differentiation over culturing time, and thus for both conditions wild type and *capn3*-deficient (not shown) mice. As a result, in subsequent single-cell Ca^2+^ measurements myoblasts and myotubes were probed independently as they exhibit different differentiation states.

In a second set of experiments we analysed *capn3* expression in myoblasts and myotubes from normal and *capn3*-deficient mice. RT-PCR was carried out with RNA extracted from the myoblast and myotube differentiation stages and with specific primers covering in particular the regions of *capn3* alternative splicing [4]. Only weak expression was detected on *capn3*-deficient cells (data not shown; [6]) whereas it was present in normal cells at both stages. In addition, the differentiation process in this cell culture system was accompanied by a change in the expression pattern of calpain 3 RNA isoforms with alternative splicing forms mostly expressed in immature cells (see MB lanes in Figure 1(b) and Table 1 for semiquantification).

During the excitation-contraction coupling phenomenon, RyR releases Ca^2+^ in response to depolarization of the plasma membrane. Previous publications reported the cleavage of RyR by a 94 kDa thiol protease into two fragments (375 kDa and 150 kDa fragments) [7, 15]. This cleavage results in an enhancement of Ca^2+^ efflux from SR vesicles. We examined this protein by western blot in normal and *capn3*-deficient whole skeletal muscle samples (Figure 1(c) and Table 2 for semiquantification). The cleaved fragments were obtained in both samples indicating that calpain 3 is not necessary for cleavage in whole muscle and that it most likely cannot be involved in the results dealing with the Ca^2+^ release from RyR in cultivated proliferating satellite cells.

Deficiency in calpain 3 is known to be associated with apoptosis [5] as indicated by increases of caspase 3 activity [16]. Analysis of the cleavage of a fluorescent substrate specifically by caspase 3 was performed in cells at the myoblast stage. Figure 1(d) highlights the significant raise of caspase 3 activity in calpain 3-deficient cells in comparison to wild type cells. Caspase 3 being a marker for apoptosis, this result therefore suggests a probable increase of the apoptotic rate when calpain 3 is lacking in mouse myoblasts. 

### 3.2. Caffeine-Induced [Ca^2+^]_i_ Increase in Isolated Myoblasts and Myotubes

**Figure 2:**
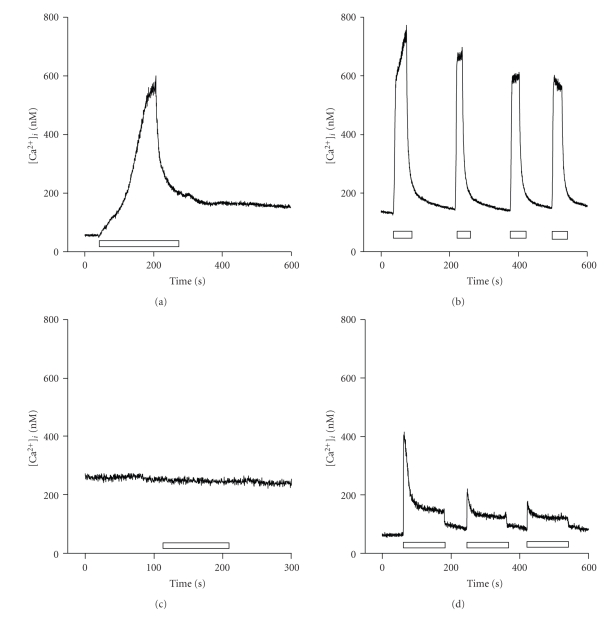
Effect of caffeine on [Ca^2+^]*_i_* in isolated myoblasts and myotubes. Representative traces show the typical time course of the response to 20 mM caffeine observed in (a) wild type (+/+) myoblast; (b) wild type (+/+) myotube; (c) *capn3*-deficient (−/−) myoblast; (d) *capn3*-deficient (−/−) myotube. The duration of drug exposure is represented (open bars).

**Figure 3:**
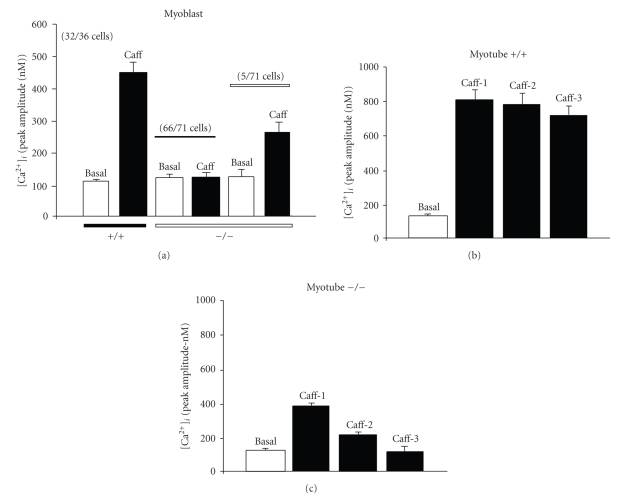
Peak amplitude of [Ca^2+^]*_i_* response of myoblasts and myotubes to caffeine. Bar diagrams summarize the response of the cell types shown in Figure 2. (a) myoblasts (+/+ and −/−): single application of 20 mM caffeine; (b) myotubes (+/+); (c) myotubes (−/−): 3 successive applications of 20 mM caffeine. The number of cells pooled in a category and the total number of cells tested are given in brackets.

Caffeine, the best known agonist of ryanodine receptors [17], was used at 20 mM throughout the study to ensure the complete activation of the RyRs of the sarcoplasmic reticulum (SR) [18, 19]. [Ca^2+^]*_i_* release by caffeine was tested on myoblasts and myotubes cultured from wild type (+/+) and *capn3*-deficient (−/−) mice (Figure 2). Resting [Ca^2+^]*_i_* levels in cell types of both +/+ and −/− mice did not change significantly (103 ± 4 nM; *n* = 187). After local caffeine application, +/+ myoblasts showed a single and slow increase in [Ca^2+^]*_i_*, that peaked after 3 minutes of caffeine application and decayed slowly, even before the caffeine was washed out (Figure 2(a)) presumably confirming the presence at this stage of the Na^+^/Ca^2+^ exchanger at the plasma membrane level [20]. The number of cells responding to caffeine in these cell types is summarised in Figure 3(a). Thirty-two out of 36 +/+ myoblasts showed an increase in [Ca^2+^]*_i_* after the application of caffeine. No [Ca^2+^]*_i_* response to caffeine was observed for repeated applications (data not shown) even when the caffeine was quickly taken off. By contrast, +/+ myotubes exhibited a constant, repeated and reproducible increase in [Ca^2+^]*_i_* after brief (30s) and repeated applications of caffeine (Figure 2(b)). The mean peak amplitude of the [Ca^2+^]*_i_* response is plotted in Figure 3(b). In contrast, −/− myoblasts failed to exhibit the [Ca^2+^]*_i_*-induced response to caffeine in a huge majority of cells (Figure 2(c) and Figure 3(a); 66 cells out of 71). A very small response was observed in only 5 out of 71 cells (Figure 3(a)). In contrast to +/+ myotubes, a progressive desensitization of the [Ca^2+^]*_i_* response to repeated caffeine applications (3 min each) was observed in −/− myotubes (Figure 2(d)). In addition, the peak amplitude of the responses induced by 3 successive shots of 20 mM caffeine observed in −/− myotubes was significantly lower than in wild-type: a 60%, 75% and 90% decrease, respectively (*n* = 12; Figure 3(c) versus Figure 3(b)).

### 3.3. Effect of Caffeine and CPA on [Ca^2+^]_i_ in Isolated Myoblasts and Myotubes from Wild Type (+/+) and capn3-Deficient (−/−) Mice

**Figure 4:**
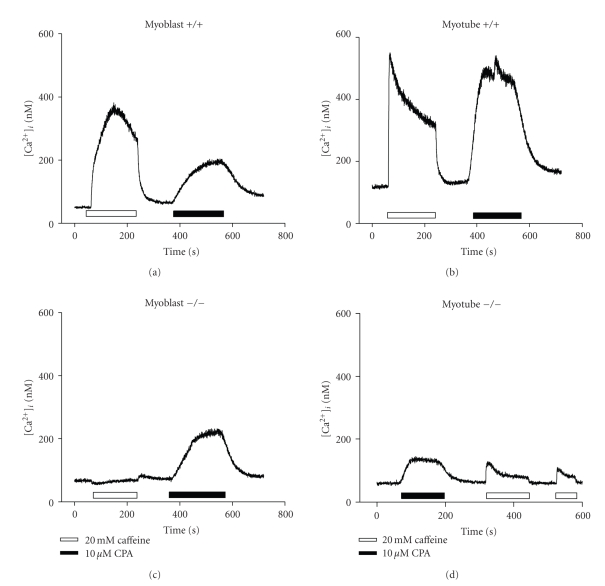
Effect of caffeine and CPA on [Ca^2+^]*_i_* in isolated myoblasts and myotubes. Representative traces show the typical time course of the response to 20 mM caffeine and 10 *μ*M CPA observed in (a) wild type (+/+) myoblast; (b) wild type (+/+) myotube; (c) calpain 3-deficient (−/−) myoblast; (d) calpain 3-deficient (−/−) myotube. The duration of exposure to caffeine (open bars) and CPA (gray-dashed bars) is represented.

**Figure 5:**
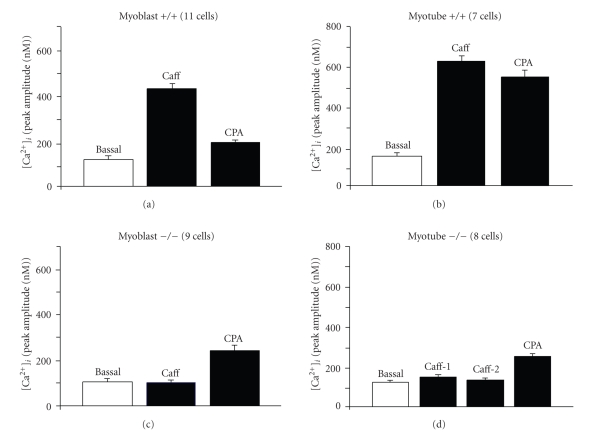
Peak amplitude of [Ca^2+^]*_i_* response of myoblasts and myotubes to caffeine and CPA. Bar diagrams summarize the response of the cell types shown in Figure 4. (a) myoblasts (+/+); (b) myotubes (+/+); (c) myoblasts (−/−): application of 20 mM caffeine followed by 10 *μ*M CPA; (d) myotubes (−/−):  2 successive applications of 20 mM caffeine followed by 10 *μ*M CPA. The number of cells tested is given in brackets.

We then compared the effects of CPA to those obtained with caffeine. CPA is a potent inhibitor of the sarcoplasmic reticulum (SR) Ca^2+^-ATPase [21, 22] and induces Ca^2+^ mobilization from internal Ca^2+^ stores, preferentially depleting InsP_3_-sensitive stores [23]. In +/+ myoblasts CPA (10 *μ*M) induced an increase of [Ca^2+^]*_i_* of much lower amplitude (Figure 4(a), right peak) than when induced by a prior exposure to caffeine (Figure 4(a); left peak). Tested on +/+ myotubes, both caffeine and CPA induced a similar [Ca^2+^]*_i_* increase, with a peak amplitude significantly higher (Figure 4(b)) than the one observed in myoblasts for both drugs (Figure 4(a)). The differences found between the responses to caffeine and CPA in +/+ myoblasts and +/+ myotubes was found to be significant (*P* < 0.05; Figures 5(a) and 5(b). 

Interestingly, in *capn3*-deficient cells (−/−), the [Ca^2+^]*_i_* responses to caffeine and CPA were quite different (Figures 4(c) and 4(d) for the parameters tested. First of all, as already illustrated in Figure 2(c), the Ca^2+^ response to caffeine in −/− myoblasts was totally absent (Figure 4(c) left trace), and of small amplitude in −/− myotubes (Figure 4(d) right trace). By comparison CPA induced a significant [Ca^2+^]*_i_* rise in −/− myoblasts (Figure 4(c) right peak), but a much smaller increase in −/− myotubes, even when CPA was applied before caffeine (Figure 4(d) left peak). The peak amplitude of the various [Ca^2+^]*_i_* responses was quantified and are summarised in Figure 5. 

### 3.4. Influence of the External Ca^2+^ Concentration and of Ca^2+^ Channels at the Plasma Membrane on SR Ca^2+^ Release

The [Ca^2+^]*_i_* responses induced by caffeine were also tested in low Ca^2+^ EGTA-buffer to investigate the dependence upon external Ca^2+^ in myotubes obtained from wild type and *capn3*-deficient (−/−) mice. Indeed, the removal of extracellular Ca^2+^ did not significantly affect the [Ca^2+^]*_i_* responses induced by 20 mM caffeine on +/+ myotubes (results not shown). However, a major reduction in the [Ca^2+^]*_i_* response to caffeine was observed in −/− myotubes, in the absence of extracellular Ca^2+^ (results not shown). 

**Figure 6:**
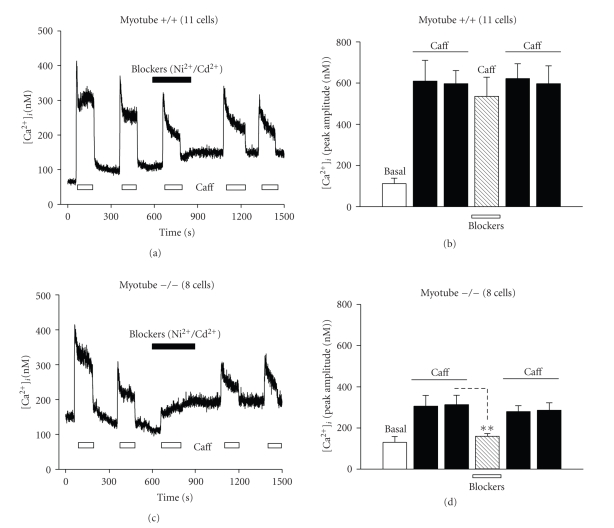
Effect of caffeine on [Ca^2+^]*_i_* in isolated myotubes in the presence of Ca^2+^ channel blockers (Ni^2+^/Cd^2+^). Representative traces show the typical time course of the response to 20 mM caffeine observed in (a) wild type (+/+) myotube; (c) calpain 3-deficient (−/−) myotube, each treated with Ni^2+^/Cd^2+^. The duration of exposure to caffeine (open bars), or 50 *μ*M Ni^2+^/100 *μ*M Cd^2+^ (closed bars) is represented. Bar diagrams (b) and (d) summarize the peak amplitude of the [Ca^2+^]*_i_* response of myotubes to caffeine and CPA. The responses from the wild type myotubes (+/+; b) and calpain 3-deficient myotubes (−/−; d), are shown. Fifty *μ*M Ni^2+^/100 *μ*M Cd^2+^ was added to the extracellular medium prior to the second application. The number of cells tested is given in brackets.

The next experimental design aimed to dissect the functional interaction between RyR and Ca^2+^ channels of the plasma membrane in myoblasts and myotubes of wild type and *capn3*-deficient (−/−) mice. In this respect, we focused our study on myotubes, because −/− myoblasts appeared insensitive to caffeine. We subjected myotubes to successive exposures to caffeine, in the absence or presence of nonspecific Ca^2+^ channel blockers (i.e., a mixture of 100 *μ*M Cd^2+^ and 50 *μ*M NiCl_2_). None of the Ca^2+^ channel blockers affected the [Ca^2+^]*_i_* response induced by caffeine in +/+ myotubes (Figures 6(a) and 6(b)). In sharp contrast, Ca^2+^ channel blockers significantly reduced the [Ca^2+^]*_i_* response induced by caffeine in −/− myotubes (Figures 6(c) and 6(d)). It is noteworthy that, at the end of the inhibition of Ca^2+^ entry (Figures 6(a) and 6(c)), the baseline increased further suggesting that there was a Ca^2+^ re-entry in both types of cells. However, the relative amplitudes of the 4th and 5th caffeine applications were still different between +/+ and −/− myotubes. Moreover, after starting the inhibition of Ca^2+^ entry (Figure 6(c)), the baseline decreased further suggesting that there was constitutive Ca^2+^ entry in −/− myotubes. In addition, in other set of experiments, the effects of various more specific blockers were examined, among them the most important Ca^2+^ channel sub-type blockers, such as nicardipine (L-type blocker), omega GVIA-N-type, omega MVIIC/MVIIA-a P/Q type, omega-Aga-IVA (a P/Q-type blocker), and SNX-482 (a R-type blocker). Interestingly, only nicardipine at 800 nM significantly blocked the Ca^2+^ response induced by caffeine in −/− myotubes (control: 568 ± 32 nM; nicardipine: 102 ± 25 nM; *n* = 4; *P* < 0.05) suggesting that the response could not be directly mediated by caffeine-sensitive channels. 

## 4. Discussion

Calpains are a family of Ca^2+^-dependent cysteine proteases (for review articles, see [1–3]), the members of which are expressed ubiquitously (calpains 1 and 2) or in a tissue-specific way (calpain 3 is skeletal muscle specific and an isoform of calpain 3 was found in the lens). In addition to Ca^2+^ ions, the activation of ubiquitous calpains can be modulated by association with a 30 kD small sub-unit, or with membranes, by the autolysis of the N-terminus, or by calpastatin, a specific inhibitor. Their function in muscle has received increasing interest because of the finding that the activation and concentration of the ubiquitous calpains were found to be increased in the mouse model of Duchenne muscular dystrophy (mdx mice). Moreover, protein degradation was enhanced in mdx muscle [24], and it was argued that increased degradation resulted from the elevated Ca^2+^ levels existing within the dystrophic muscle. Possible substrates of calpains are the membrane-associated cytoskeletal proteins, the plasma membrane Ca^2+^-ATPase, and the ion channel proteins. Interestingly, the Ca^2+^ pump located in the plasma membrane is a preferred substrate of calpain in erythrocytes [25]. If impaired in dystrophin-deficient muscle, this calpain action would, in addition to provoking an excess of Ca^2+^ influx, disturb an important extrusion pathway. Calpains in normal tissue supposedly exert regulatory roles. It is therefore assumed that in the dystrophic process, a deficiency in one of the calpains would result in affecting a metabolic pathway rather than muscle proteolysis. Calpains cleave substrates at restricted locations [3] and are unlikely to be involved in mediating major house-keeping degradative functions. Thus, current evidence supports a role for pathologically-high calpain activity in muscular dystrophy through the disruption of specific regulatory mechanisms in muscle, rather than through an increase in nonspecific proteolysis.

In this study we have used mouse primary cultures of skeletal muscle from normal and *capn3* −/− mice recently generated at Genethon by I. Richard's laboratory. *Capn3*-deficient mice are fully fertile and viable and show a mild muscular dystrophy that affects a specific group of muscles. Interestingly, affected muscles manifest a similar apoptosis-associated perturbation of the I{kappa}B{alpha}/NF-{kappa}B pathway as seen in LGMD2A patients [5] and *capn3*-deficient mice [6].

The availability of primary cultures of skeletal muscle cells from normal and *capn3* −/− mice provides an opportunity to tackle in the near future the upstream and downstream events occurring during a pharmacologically-induced [Ca^2+^]*_i_* rise in myoblasts and myotubes from normal and *capn3* −/− mice. Notably, it is possible that calpain 3 acts as a feedback regulator for calcium homeostasis in skeletal muscle cells by exerting an action on RyR. The latter, also known as the Ca^2+^ release channel of the SR, is a key protein involved in excitation-contraction coupling. Its activity is regulated by a 94 kDa thiol-protease of the junctional SR membranes which specifically cleaves one site on RyR. This cleavage results in enhanced Ca^2+^ efflux from SR vesicles [7, 15].

Importantly, in our hands calpain 3 seemed not to be necessary for cleavage in skeletal muscles in vitro. The cleavage of RyR in the absence of calpain 3 (muscle-specific) could be due to calpain 1 and/or 2, which are widely expressed in all cell types. The activity of these calpains could indeed be redundant in that case. To assess the activity of ryanodine-sensitive internal Ca^2+^ stores, we applied caffeine stimulations, caffeine being a well-known potent RyR activator [17]. 

The effectiveness of caffeine in normal myotubes in comparison to myoblasts indicates a maturation of proper RyR signalling in culture during the fusion process. Importantly, both myoblasts and myotubes from *capn3* −/− mice displayed weaker amplitudes of the caffeine-induced [Ca^2+^]*_i_* transients than in normal cells, which could indicate a lower SR Ca^2+^ loading state in the KO skeletal cells, a decreased number of RyR at the SR membrane surface, or a decreased sensitivity of these receptors but independently of any cleavage by calpain 3.

While CPA, a compound that depletes internal Ca^2+^ stores [26], evoked increases of [Ca^2+^]*_i_* under all conditions tested, it appeared that those responses were weaker in* capn3* −/− myotubes in comparison to wild-type myotubes, reinforcing the hypothesis that SR Ca^2+^ loading decreased in *capn3* −/− myotubes or indicating that SR Ca^2+^-ATPases were less expressed or less sensitive to the blocker in KO myotubes. The difference in CPA responses between wild-type myoblasts and myotubes is most likely due to a change in the size of the SR that correlates with a change in the size of the cells during fusion in culture, myotubes having a larger area. 

Caffeine is thought to directly activate RyR at the SR membrane, leading to the opening of this channel and the release of Ca^2+^ from the SR into the cytosol, independently of any Ca^2+^ influx through the plasma membrane. The fact that low extracellular Ca^2+^ and blockers of LCCs abolished caffeine-induced [Ca^2+^]*_i_* increases in −/− myotubes suggests that RyR opening and SR Ca^2+^ release lead to a Ca^2+^ influx through LCCs solely in the KO myotubes. Since cytosolic Ca^2+^ is known to negatively regulate these channels (inactivation) and that the release from the SR is not sufficient to evoke a depolarization enabling the opening of LCCs [27], it is most likely that an additional channel at the plasma membrane induces a depolarization in response to the caffeine-evoked [Ca^2+^]*_i_* increases in −/− myotubes.

Finally, another possibility would be that the lack of calpain 3 leads to a decrease of RyR sensitivity to caffeine, probably involving a regulation of the post-translational maturation of the receptor, and thus independently of any functional cleavage of RyR. It is noteworthy that RyR contains many endogenous cysteines in the cytoplasmic domain of the protein. Hence the binding of caffeine to its site on the cytosolic face of RyR would require an activation of RyR by extracellular Ca^2+^ entry in order to induce the proper opening of the receptor. 

Taken together, the results obtained in −/− myotubes indicate: (i) a decrease of the SR load, (ii) an alteration of RyR signalling, (iii) an increase of LCC activity (i.e., constitutive Ca^2+^ entry), but (iv) no increase of the basal intracellular Ca^2+^ concentration. As the major system of Ca^2+^ extrusion from the cytosol is the Na^+^/Ca^2+^ exchanger, it is very tempting to speculate that the activity of the exchangers could be increased (higher protein expression and/or higher rate of ion flow) in KO myocytes to maintain the level of Ca^2+^ in the cells. However, this will be the subject for another specific investigation.

In conclusion, by using a knockout strategy, we could induce a skeletal muscle dystrophy in mice due to the absence of calpain 3 and thus draw a general picture of the cellular pathways involved in this disease. The LGMD2A dystrophy is characterised by (i) an increase of caspase 3 activity, (ii) a deregulation of the I{kappa}B{alpha}/NF-{kappa}B pathway leading to apoptosis [5, 16], (iii) an increase of membrane permeability, (iv) a decrease in the size of the SR and v) a dysfunction of RyR signalling. Indeed, our pharmacological study sheds more light on the mechanism of Ca^2+^ remodelling in the failing skeletal muscle, and we propose a major regulatory role for *capn3* on SR Ca^2+^ release, probably mediated by an increased participation of LCC in Ca^2+^ entry to compensate for the alteration of SR functionality. Moreover, calpain-dependent proteolysis might be involved not only in the regulation of RyR channels themselves, but also in the activation by splitting of RyR auxiliary proteins forming the RyR1 multi-protein complex.

Of interest, recent work from our group showed that not calpain 3, but *μ*-calpain is important for the phenomenon of excitation-contraction (Ca^2+^-induced) uncoupling in normal and *capn3* −/− skeletal muscle fibres [28], suggesting that the pattern of activities and functions of the different calpains is quite complex and that each calpain seems to play a very specific and defined role in the regulation of Ca^2+^ signalling. 

## Figures and Tables

**Table 1:** Quantification of the expression of calpain 3 transcripts in wild type myoblasts and myotubes. These data were obtained from the gel electrophoresis of the RT-PCR reactions using the primer pairs p94sys3, p94sys5 and p94sys6 on murine myoblast (MB) or myotube (MT) mRNA (see Figure 1(b)). The gel and the corresponding quantification are representative of 3 different experiments where similar results were observed. These results have to be taken qualitatively since the experiments were performed by classical RT-PCR and not by quantitative RT-PCR. Numbers are given in arbitrary units.

Primer	p94sys3		p94sys5		p94sys6	
Cell type	MB	MT	MB	MT	MB	MT
Band 1	43596	165378	155681	205283	66937	46845
Band 2	207433	89706	73219	69370	45719	
Band 3					43022	
Total	251029	255084	228900	274653	155678	46845

**Table 2:** Western blot quantification: cleavage of the ryanodine receptor in wild type and calpain 3-deficient skeletal muscles. The gel presented in Figure 1(c) was analysed in terms of band intensities (numbers are given in arbitrary units). The lower band is 58% of the higher band (fixed to 100%) in lane 2, and 51% in lane 3. The amount of the second band is related to small degradation condition (time and temperature), but is always identical in the two situations wild type and calpain 3-deficient skeletal muscles. The blot and the corresponding quantification are representative of at least 3 independent experiments showing no difference between the two types of mice.

Sample	1 = human skeletal muscle	2 = *capn3* −/− mouse skeletal muscle	3 = wild type mouse skeletal muscle
Band 1	90982	69472	52878
Band 2	92773	32041	38331
Band 3	49076		
Total	232831	101513	91209
